# Knowledge of Palestinian women about cervical cancer warning signs: a national cross- sectional study

**DOI:** 10.1186/s12889-021-11792-8

**Published:** 2021-10-02

**Authors:** Mohamedraed Elshami, Ibrahim Al-Slaibi, Hanan Abukmail, Mohammed Alser, Afnan Radaydeh, Alaa Alfuqaha, Mariam Thalji, Salma Khader, Lana Khatib, Nour Fannoun, Bisan Ahmad, Lina Kassab, Hiba Khrishi, Deniz Elhussaini, Nour Abed, Aya Nammari, Tumodir Abdallah, Zaina Alqudwa, Shahd Idais, Ghaid Tanbouz, Ma’alem Hajajreh, Hala Abu Selmiyh, Zakia Abo-Hajouj, Haya Hebi, Manar Zamel, Refqa Skaik, Lama Hammoud, Siba Rjoub, Hadeel Ayesh, Toqa Rjoub, Rawan Zakout, Amany Alser, Nasser Abu-El-Noor, Bettina Bottcher

**Affiliations:** 1 Harvard Medical School, 25 Shattuck Street, Boston, MA 02115 USA; 2 Ministry of Health, Gaza, Palestine; 3 Almakassed Hospital, Jerusalem, Palestine; 4 Faculty of Medicine, Islamic University of Gaza, Gaza, Palestine; 5 Faculty of Medicine, Al-Quds University, Jerusalem, Palestine; 6 Faculty of Graduate Studies, An-Najah National University, Nablus, Palestine; 7 Faculty of Medicine, An-Najah National University, Nablus, Palestine; 8 Faculty of Pharmacy, Alazhar University of Gaza, Gaza, Palestine; 9 Faculty of Dentistry and Dental Surgery, Al-Quds University, Jerusalem, Palestine; 10 Faculty of Medicine, Alazhar University of Gaza, Gaza, Palestine; 11 Alia Hospital, Hebron, Palestine; 12 Al-Shiffa hospital, Gaza, Palestine; 13 Faculty of Nursing, Islamic University of Gaza, Gaza, Palestine

**Keywords:** Cervical cancer, Early detection, Survival, Symptom, Warning sign, Awareness, Knowledge, Early presentation, Palestine

## Abstract

**Background:**

Timely presentation and diagnosis of cervical cancer (CC) are crucial to decrease its mortality especially in low- and middle-income countries like Palestine. This study aimed to evaluate the knowledge of Palestinian women about CC warning signs and determine the factors associated with good knowledge.

**Methods:**

This was a national cross-sectional study conducted between July 2019 and March 2020 in Palestine. Stratified convenience sampling was used to recruit adult women from hospitals, primary healthcare centers, and public spaces of 11 governorates. A translated-into-Arabic version of the validated CC awareness measure (CeCAM) was used to assess women’s knowledge of 12 CC warning signs.

**Results:**

Of 8086 approached, 7223 participants completed the CeCAM (response rate = 89.3%). A total of 7058 questionnaires were included in the analysis: 2655 from the Gaza Strip and 4403 from the West Bank and Jerusalem (WBJ). The median age [interquartile range] for all participants was 34.0 [24.0, 42.0] years. Participants recruited from the WBJ were older, getting higher monthly income, and having more chronic diseases than those recruited from the Gaza Strip.

The most frequently identified warning sign was ‘vaginal bleeding after menopause’ (*n* = 5028, 71.2%) followed by ‘extreme generalized fatigue’ (*n* = 4601, 65.2%) and ‘unexplained weight loss’ (*n* = 4578, 64.9%). Only 1934 participants (27.4%) demonstrated good knowledge of CC warning signs. Participants from the Gaza Strip were slightly more likely than participants from the WBJ to have a good level of knowledge. Factors associated with having good knowledge included having a bachelor or postgraduate degree, being married, divorced, or widowed as well as knowing someone with cancer.

**Conclusion:**

The overall awareness of CC warning signs was low. Educational interventions are needed to increase Palestinian women’s awareness of CC warning signs.

**Supplementary Information:**

The online version contains supplementary material available at 10.1186/s12889-021-11792-8.

## Background

Cervical cancer (CC) is the most common gynecological cancer worldwide [1, 2]. The global annual deaths related to CC are over 300,000 with half of these deaths occurring in countries with low and medium human development indices [2]. In a recent international report, the age-standardized incidence and mortality rates of CC were 13.3 and 7.3 per 100,000 females, respectively [2]. In the Eastern Mediterranean region, the estimated age-standardized incidence and mortality rates of CC were 5.3 and 3.4 per 100,000 females, respectively [3]. In 2018, Palestine had a relatively low age-standardized incidence rate of 2.5 per 100,000 females. However, Palestine had a higher age-standardized mortality rate of 1.9 per 100,000 females than some other countries in the region such as Iraq (1.3 per 100,000 females), Yemen (1.4 per 100,000 females), Saudi Arabia (1.5 per 100,000 females), and Jordan (1.8 per 100,000 females) [4].

CC is one of the most preventable and treatable cancers especially if the premalignant lesions are detected and treated early before progressing to malignancy [5]. Survival rates of CC also vary depending on the stage at the time of diagnosis with better prognosis among women diagnosed with early-stage disease [6–8]. Therefore, early diagnosis is crucial to decrease mortality related to CC. Several factors were reported to play a role in delaying the diagnosis of CC including low awareness of CC warning signs, limited access to healthcare services, and emotional barriers to seek medical advice (e.g., feeling scared) [9–12].

In Palestine, there are no screening programs for CC or vaccination program for the main cause of CC, human papillomavirus (HPV) [13]. This further increases the importance of determining women’s knowledge of CC warning signs as it may impact their decision to visit doctors [12, 14]. In addition, assessment of the existing awareness of CC warning signs will help to guide future educational interventions aiming to increase public awareness [14, 15]. Greater public awareness of CC warning signs may lead to shortening the time to seek medical advice, which in turn facilitates early detection of CC and increases survival rates [16–18]. This is especially important where no screening programs for CC exist as in Palestine.

This national study aimed to: (i) assess the women’s level of knowledge of CC warning signs in the Palestinian community, (ii) compare this knowledge among women recruited from the Gaza Strip vs. the West Bank and Jerusalem (WBJ), and (iii) determine the factors associated with good knowledge of CC warning signs.

## Methods

### Study design, population, and settings

A national cross-sectional study was conducted from July 2019 to March 2020 in Palestine. Adult Palestinian women were the target population and were recruited to participate in this study from hospitals, primary healthcare centers (PHCs), and public spaces. Governmental hospitals and PHCs are the main sites for providing healthcare services in Palestine and are distributed in two main geographical areas: (i) the Gaza Strip and (ii) the WBJ. Governmental general hospitals with a bed capacity of more than 100 and PHCs with level four services (i.e., providing all primary healthcare services) were eligible. Public spaces in the same governorates of hospitals and PHCs were also involved. These included markets, downtowns, mosques, churches, parks, malls, and restaurants.

In 2020, the unemployment rate of Palestinian women was 40.1% (46.6% in the Gaza Strip vs. 15.7% in the WBJ) [19]. In 2021, 1,454,846 women are 18 years or over, representing 27.9% of the total population of 5,222,748 [20]. Palestinian adult women (aged 18 or older), attending one of the data collection sites, were invited to participate. Participants were excluded if they were holding a citizenship other than Palestinian, visiting the oncology departments, and working or studying in a health-related field.

### Sampling methods

The Palestinian MoH has 43 hospitals; 29 of them are in the West Bank and 14 are in the Gaza Strip. There are 11 general MoH hospitals with a bed capacity of more than 100; six in the West Bank and five in the Gaza Strip [17]. Jerusalem has no hospitals that belong to the MoH. However, non-governmental organizations (NGOs) own two general hospitals with a bed capacity of more than 100. The Palestinian MoH also has 475 PHCs. Among them, 26 are level four: 17 are in the WBJ and nine in the Gaza Strip [17]. In 2019, the estimated female population aged 15 years or older in the WBJ was 947,100 females while that in the Gaza Strip was 587,271 females (ratio 1:1.6) [21]. Therefore, stratified convenience sampling was used to achieve a similar ratio in the two regions and participants were recruited from 11 hospitals, 12 PHCs, and public spaces in 11 out of 16 governorates of Palestine: seven in the WBJ and four in the Gaza Strip.

### Questionnaire and data collection

The Cervical Cancer Awareness Measure (CeCAM), which is a validated standardized questionnaire developed to measure the awareness of CC in the general population, was used [8]. The questionnaire consisted of two sections. The first section included socio-demographic questions such as age, menarche, highest level of education, occupation, monthly income, marital status, place of residency, having a chronic disease, and knowing someone with cancer. The second section comprised of one question based on a 4-point Likert scale (1 = not at all confident, 4 = very confident) to ask the participants about their confidence on noticing possible CC warning signs and 12 questions using a 5-point Likert scale (1 = strongly disagree, 5 = strongly agree) to assess their knowledge of CC warning signs.

To minimize the possibility of participants answering questions randomly, the questions in the original CeCAM with yes/no/unknown responses were modified into 5-point Likert scale questions. Meanwhile, the participants’ responses were converted to correct/incorrect responses similar to what was done in previous studies [11, 12]. The sign of ‘extreme generalized fatigue’ was added to the questionnaire since it was mentioned in other forms of the Cancer Awareness Measure [22, 23], and it was thought that it would be helpful to include it in the context of CC.

The questionnaire passed through the process of translation and adaptation of instruments recommended by the World Health Organization [24]. It was translated from English to Arabic by two bilingual healthcare professionals and then back-translated into English by another two bilingual healthcare professionals who had relevant clinical and research experiences in gynecology, public health, and survey design. A pilot study was conducted with 130 respondents to test the clarity of the questions of the Arabic CeCAM. These were not included in the final study. Internal consistency was measured using Cronbach’s Alpha, which reached an acceptable value (α =0.816).

Participants were invited to face-to-face interviews for the completion of the questionnaire. Data were collected utilizing the secure, user-friendly data collection tool ‘Kobo Toolbox’ which is accessed via smartphones [25]. It allowed using a pre-designed data collection sheet with tick boxes and dropdown menus for easy and quick data collection and entry. Female data collectors with a medical background were trained on how to use the electronic tool and how to recruit participants, approach them, and facilitate completion of the questionnaire.

### Statistical analysis

Descriptive statistics were utilized to summarize participant characteristics. For continuous non-normally distributed variables, the median and interquartile range were used to describe them. Categorical variables were summarized using frequencies and percentages. Age was categorized into three groups to reflect the age-associated risk of CC (21–40 years) [8]. The minimum wage in Palestine is 1450 NIS, which is about $450 [26]. Therefore, it was used to divide the participants in terms of their monthly income into two groups. Baseline characteristics of participants from the Gaza Strip vs. the WBJ were compared using Kruskal-Wallis test if they were continuous or using Pearson’s Chi-square test if they were categorical.

Frequencies and percentages were used to describe the confidence of participants to detect possible CC warning signs with a comparison being made using Pearson’s Chi-Square test. As for recognizing CC warning signs, answering with ‘strongly agree’ or ‘agree’ was considered as a correct answer, while answering with ‘strongly disagree’, ‘disagree’, or ‘not sure’ was considered as an incorrect answer. CC warning signs were categorized into three categories: signs with blood, signs with pain, and signs of a non-specific nature. Frequencies and percentages were utilized to describe the recognition of each of the CC warning signs with comparisons being performed using Pearson’s Chi-Square test. Then, bivariable and multivariable logistic regression analyses were used to test the association between recognizing each warning sign and participant characteristics. Results of the bivariable analyses are provided in the supplementary materials, please see Additional file 1. The model of the multivariable analysis included all participants and adjusted for the following variables: age-group, educational level, occupation, monthly income, place of residency, marital status, having a chronic disease, knowing someone with cancer, and site of data collection. The model was pre-specified based on previous studies [8, 27–29].

To evaluate the participants’ knowledge level of CC warning signs, a scoring system was adopted from previous studies [11, 18]. Each correct answer was given one point. The total score was calculated and ranged from 0 to 12. It was then categorized into three categories: poor knowledge (0 to 4), fair knowledge (5 to 8), and good knowledge (9 to 12). A comparison in the knowledge level between the Gaza Strip vs. the WBJ was made using Pearson’s Chi-Square test. The association between participant characteristics and having a good level of knowledge was tested using bivariable and multivariable logistic regression with the same model mentioned above. Missing data were completely random and were handled using complete case analysis. Data were analyzed using Stata software version 15.0 (StataCorp, College Station, Texas, United States).

## Results

### Characteristics of participants

Of the 8086 participants approached, 7223 completed the questionnaire (response rate = 89.3%). A total of 7058 questionnaires was included in the analysis (30 did not meet inclusion criteria and 135 had missing values); 2655 from the Gaza Strip and 4403 from the WBJ. The median age [IQR] for all participants was 32.0 years [24.0, 42.0] (Table 1). Participants recruited from the WBJ were older, getting higher monthly income, and having more chronic diseases than those recruited from the Gaza Strip.

**Table 1:** Characteristics of study participants

Characteristic	Total (***n*** = 7058)	Gaza Strip (***n*** = 2655)	WBJ (***n*** = 4403)	***p***-value
**Age**, median [IQR]	32 [24, 42]	30 [24, 39]	33 [24, 44]	< 0.001
**Age group**, n (%)				
18 to 20	756 (10.7)	249 (9.4)	507 (11.5)	< 0.001
21 to 40	4331 (61.4)	1809 (68.1)	2522 (57.3)	
41 or older	1971 (27.9)	597 (22.5)	1374 (31.2)	
**Educational level**, n (%)				
Illiterate	127 (1.8)	37 (1.4)	90 (2.0)	< 0.001
Primary	409 (5.8)	127 (4.8)	282 (6.4)	
Preparatory	1064 (15.1)	378 (14.2)	686 (15.6)	
Secondary	2293 (32.5)	955 (36.0)	1338 (30.4)	
Diploma	766 (10.9)	303 (11.4)	463 (10.5)	
Bachelor	2261 (32.0)	817 (30.8)	1444 (32.8)	
Postgraduate	138 (1.9)	38 (1.4)	100 (2.3)	
**Occupation**, n (%)				
Housewife	4647 (65.8)	2008 (75.6)	2639 (59.9)	< 0.001
Employed	1476 (20.9)	348 (13.1)	1128 (25.6)	
Retired	69 (1.0)	11 (0.4)	58 (1.3)	
Student	866 (12.3)	288 (10.9)	578 (13.2)	
**Monthly income ≥ 1450 NIS**, n (%)	4666 (66.1)	693 (26.1)	3973 (90.2)	< 0.001
**Having a chronic disease**, n (%)	1397 (19.8)	417 (15.7)	980 (22.3)	< 0.001
**Knowing someone with cancer**, n (%)	4083 (57.9)	1483 (55.9)	2600 (59.1)	< 0.001
**Marital status**, n (%)				
Single	1657 (23.4)	527 (19.8)	1130 (25.6)	< 0.001
Married	5058 (71.7)	2025 (76.3)	3033 (68.9)	
Divorced	154 (2.2)	45 (1.7)	109 (2.5)	
Widowed	189 (2.7)	58 (2.2)	131 (3.0)	
**Site of data collection, n (%)**				
Public spaces	2695 (38.2)	863 (32.5)	1832 (41.7)	< 0.001
Hospitals	1890 (26.8)	642 (24.2)	1248 (28.3)	
Primary healthcare centers	2473 (35.0)	1150 (43.3)	1323 (30.0)	

*n* number of participants, *IQR* interquartile range, *WBJ* West Bank and Jerusalem

### Confidence and recognition of CC warning signs

Only 2122 participants (30.0%) felt confident to notice a possible CC warning sign. Participants from the Gaza Strip were more likely to have confidence than participants from the WBJ (33.9% vs 27.7%). Warning signs with blood were the most commonly recognized signs followed by signs of a nonspecific nature and those with pain (Table 2). The most frequently identified sign was ‘vaginal bleeding after menopause’ (*n* = 5028, 71.2%) followed by ‘extreme generalized fatigue’ (*n* = 4601, 65.2%) and ‘unexplained weight loss’ (*n* = 4578, 64.9%). Those warning signs were also the most identified signs in both the Gaza Strip and WBJ.

**Table 2:** Recognition of cervical cancer warning signs

Category of warning signs	Warning sign	Total (***n*** = 7058) n (%)	Gaza Strip (***n*** = 2655) n (%)	WBJ (***n*** = 4403) n (%)	***p***-value
**Warning signs with blood**	Vaginal bleeding after menopause	5028 (71.2)	2051 (77.3)	2977 (67.6)	< 0.001
	Vaginal bleeding between periods	4190 (59.4)	1729 (65.1)	2461 (55.9)	< 0.001
	Having menstrual periods that are heavier or longer than usual	4142 (58.7)	1648 (62.1)	2494 (56.6)	< 0.001
	Vaginal bleeding during or after sex	3684 (52.2)	1480 (55.7)	2204 (50.1)	< 0.001
	Blood in the stool or urine	3496 (49.5)	1175 (44.3)	2321 (52.7)	< 0.001
**Warning signs with pain**	Persistent pelvic pain	4188 (59.3)	1592 (60.0)	2596 (59.0)	0.41
	Unusual discomfort or pain during sex	3308 (46.9)	1285 (48.4)	2023 (45.9)	0.045
	Persistent lower back pain	2941 (41.7)	1144 (43.1)	1797 (40.8)	0.06
**Warning signs with nonspecific nature**	Extreme generalized fatigue	4601 (65.2)	1773 (66.8)	2828 (64.2)	0.029
	Unexplained weight loss	4578 (64.9)	1759 (66.3)	2819 (64.0)	0.06
	Persistent vaginal discharge that smells un-pleasant	3123 (44.3)	1137 (42.8)	1986 (45.1)	0.06
	Persistent diarrhea	1551 (22.0)	580 (21.8)	971 (22.1)	0.84

*n* number of participants, *WBJ* West Bank and Jerusalem

### Recognizing CC warning signs with blood

Women aged 21 to 40 years were less likely than younger women (i.e., 18–20 years) to recognize ‘vaginal bleeding between periods’ (OR = 0.80, 95% CI: 0.65–0.98) (Table 3). In addition, women aged ≥41 years were less likely than younger women to recognize ‘blood in the stool or urine’ (OR = 0.77, 95% CI: 0.61–0.96). Participants who were married, of higher education (i.e., bachelor and above), and were living in the Gaza Strip had a higher likelihood to identify all warning signs with blood except ‘blood in the stool or urine’. Women who knew someone with cancer were more likely than women who did not to recognize all warning signs with blood.

**Table 3:** Association between recognizing cervical cancer warning signs with blood and sociodemographic factors

Characteristic	Vaginal bleeding after menopause			Vaginal bleeding between periods			Having menstrual periods that are heavier or longer than usual			Vaginal bleeding during or after sex			Blood in the stool or urine		
	n (%)	AOR (95% CI)^a^	p-value	n (%)	AOR (95% CI)^a^	***p***-value	n (%)	AOR (95% CI)^a^	***p***-value	n (%)	AOR (95% CI)^a^	***p***-value	n (%)	AOR (95% CI)^a^	***p***-value
**Age group**															
18 to 20	551 (11.0)	Ref	Ref	463 (11.1)	Ref	Ref	398 (9.6)	Ref	Ref	301 (8.2)	Ref	Ref	444 (12.7)	Ref	Ref
21 to 40	3098 (61.6)	0.84 (0.67–1.05)	0.12	2533(60.5)	0.80 (0.65–0.98)	0.028	2523 (60.9)	1.04 (0.85–1.27)	0.70	2333 (63.3)	1.07 (0.87–1.31)	0.53	2141 (61.2)	0.82 (0.67–1.00)	0.053
41 or older	1379 (27.4)	0.91 (0.71–1.18)	0.49	1194 (28.5)	0.99 (0.78–1.24)	0.92	1221 (29.5)	1.24 (0.98–1.55)	0.07	1050 (28.5)	1.16 (0.92–1.45)	0.22	911 (26.1)	0.77 (0.61–0.96)	0.021
**Educational level**															
Illiterate	72 (1.4)	Ref	Ref	58 (1.4)	Ref	Ref	65 (1.6)	Ref	Ref	61 (1.7)	Ref	Ref	59 (1.7)	Ref	Ref
Primary	256 (5.1)	1.22 (0.80–1.84)	0.35	210 (5.0)	1.17 (0.78–1.76)	0.45	236 (5.7)	1.29 (0.86–1.93)	0.22	173 (4.7)	0.73 (0.48–1.10)	0.13	183 (5.2)	0.92 (0.61–1.38)	0.68
Preparatory	740 (14.7)	1.67 (1.13–2.46)	0.009	621 (14.8)	1.61 (1.10–2.36)	0.014	651 (15.7)	1.55 (1.06–2.26)	0.024	548 (14.9)	1.06 (0.73–1.55)	0.75	490 (14.0)	0.93 (0.64–1.36)	0.71
Secondary	1642 (32.7)	1.94 (1.32–2.83)	0.001	1350 (32.2)	1.75 (1.20–2.54)	0.004	1358 (32.8)	1.57 (1.08–2.29)	0.017	1184 (32.1)	1.25 (0.86–1.82)	0.24	1152 (33.0)	1.05 (0.72–1.52)	0.81
Diploma	553 (11.0)	2.40 (1.59–3.60)	< 0.001	454 (10.8)	2.03 (1.36–3.03)	< 0.001	431 (10.4)	1.49 (1.01–2.21)	0.047	373 (10.1)	1.24 (0.83–1.84)	0.29	354 (10.1)	0.94 (0.63–1.39)	0.76
Bachelor	1663 (33.1)	2.56 (1.73–3.80)	< 0.001	1411 (33.7)	2.38 (1.62–3.50)	< 0.001	1313 (31.7)	1.65 (1.13–2.42)	0.010	1254 (34.0)	1.78 (1.21–2.61)	0.003	1190 (34.0)	1.12 (0.77–1.64)	0.56
Postgraduate	102 (2.0)	2.98 (1.72–5.18)	< 0.001	86 (2.1)	2.54 (1.51–4.29)	< 0.001	88 (2.1)	2.02 (1.20–3.40)	0.008	91 (2.5)	2.76 (1.63–4.68)	< 0.001	68 (1.9)	0.99 (0.59–1.64)	0.96
**Occupation**															
Housewife	3361 (66.8)	Ref	Ref	2784 (66.4)	Ref	Ref	2787 (67.3)	Ref	Ref	2515 (68.3)	Ref	Ref	2226 (63.7)	Ref	Ref
Employed	1008 (20.0)	0.73 (0.63–0.86)	< 0.001	852 (20.3)	0.86 (0.75–1.00)	0.051	872 (21.1)	1.05 (0.91–1.22)	0.49	788 (21.4)	1.00 (0.87–1.16)	0.98	749 (21.4)	0.94 (0.81–1.08)	0.37
Retired	32 (0.6)	0.30 (0.18–0.50)	< 0.001	30 (0.7)	0.46 (0.28–0.77)	0.003	25 (0.6)	0.40 (0.24–0.67)	0.001	28 (0.8)	0.71 (0.42–1.20)	0.21	19 (0.5)	0.43 (0.25–0.76)	0.003
Student	627 (12.5)	0.92 (0.73–1.16)	0.48	524 (12.5)	1.02 (0.82–1.27)	0.86	458 (11.1)	1.03 (0.83–1.27)	0.82	353 (9.6)	1.01 (0.81–1.25)	0.94	502 (14.4)	1.03 (0.83–1.27)	0.80
**Monthly income**															
< 1450 NIS	1767 (35.1)	Ref	Ref	1475 (35.2)	Ref	Ref	1461 (35.3)	Ref	Ref	1285 (34.9)	Ref	Ref	1096 (31.4)	Ref	Ref
≥ 1450 NIS	3261 (64.9)	1.16 (0.99–1.35)	0.06	2715 (64.8)	1.12 (0.97–1.29)	0.11	2681 (64.7)	0.99 (0.86–1.14)	0.93	2399 (65.1)	1.02 (0.89–1.18)	0.98	2400 (68.6)	0.95 (0.83–1.09)	0.37
**Residency**															
Gaza Strip	2051 (40.8)	Ref	Ref	1729 (41.3)	Ref	Ref	1648 (39.8)	Ref	Ref	1480 (40.2)	Ref	Ref	1175 (33.6)	Ref	Ref
WBJ	2977 (59.2)	0.58 (0.50–0.67)	< 0.001	2461 (58.7)	0.64 (0.56–0.74)	< 0.001	2494 (60.2)	0.81 (0.71–0.92)	0.002	2204 (59.8)	0.82 (0.72–0.94)	0.004	2321 (66.4)	1.48 (1.30–1.69)	< 0.001
**Having a chronic disease**															
No	4027 (80.1)	Ref	Ref	3326 (79.4)	Ref	Ref	3278 (79.1)	Ref	Ref	2966 (80.5)	Ref	Ref	2842 (81.3)	Ref	Ref
Yes	1001 (19.9)	1.21 (1.04–1.40)	0.014	864 (20.6)	1.25 (1.09–1.43)	0.002	864 (20.9)	1.13 (0.99–1.30)	0.08	718 (19.5)	0.95 (0.83–1.09)	0.46	654 (18.7)	0.95 (0.83–1.08)	0.43
**Knowing someone with cancer**															
No	2005 (39.9)	Ref	Ref	1651 (39.4)	Ref	Ref	1669 (40.3)	Ref	Ref	1469 (39.9)	Ref	Ref	1367 (39.1)	Ref	Ref
Yes	3023 (60.1)	1.34 (1.21–1.50)	< 0.001	2539 (60.6)	1.25 (1.13–1.39)	< 0.001	2473 (59.7)	1.15 (1.04–1.27)	0.005	2215(60.1)	1.19 (1.07–1.31)	0.001	2129 (60.9)	1.29 (1.17–1.42)	< 0.001
**Marital status**															
Single	1147 (22.8)	Ref	Ref	943 (22.5)	Ref	Ref	857 (20.7)	Ref	Ref	625 (17.0)	Ref	Ref	911 (23.8)	Ref	Ref
Married	3645 (72.5)	1.19 (1.01–1.40)	0.033	3058 (73.0)	1.26 (1.09–1.47)	0.002	3071 (74.1)	1.38 (1.19–1.61)	< 0.001	2869 (77.9)	2.42 (2.08–2.82)	< 0.001	2404 (68.8)	0.86 (0.75–1.00)	0.051
Divorced	111 (2.2)	1.33 (0.90–1.95)	0.15	85 (2.0)	1.07 (0.75–1.51)	0.71	96 (2.3)	1.49 (1.05–2.11)	0.027	90 (2.4)	2.52 (1.78–3.57)	< 0.001	93 (2.7)	1.43 (1.00–2.02)	0.047
Widowed	125 (2.5)	1.10 (0.77–1.58)	0.60	104 (2.5)	1.08 (0.77–1.52)	0.64	118 (2.8)	1.51 (1.07–2.12)	0.019	100 (2.7)	2.48 (1.78–3.48)	< 0.001	88 (2.5)	0.90 (0.65–1.26)	0.56
**Site of data collection**															
Public spaces	1940 (38.6)	Ref	Ref	1613 (38.5)	Ref	Ref	1564 (37.8)	Ref	Ref	1352 (36.7)	Ref	Ref	1432 (41.0)	Ref	Ref
Hospitals	1296 (25.8)	0.88 (0.77–1.01)	0.07	1043 (24.9)	0.84 (0.74–0.96)	0.009	1067 (25.8)	0.89 (0.78–1.01)	0.08	983 (26.7)	1.00 (0.88–1.14)	0.99	833 (23.8)	0.78 (0.69–0.88)	< 0.001
Primary healthcare centers	1792 (35.6)	0.96 (0.84–1.10)	0.57	1534 (36.6)	1.04 (0.92–1.18)	0.52	1511 (36.5)	1.03 (0.91–1.16)	0.63	1349 (36.6)	1.03 (0.92–1.17)	0.59	1231 (35.2)	1.01 (0.90–1.14)	0.87

*AOR* adjusted odds ratio, *CI* confidence interval, *WBJ* West Bank and Jerusalem

^a^Adjusted for age-group, educational level, occupation, monthly income, marital status, residency, having a chronic disease, knowing someone with cancer, and site of data collection

### Recognizing CC warning signs with pain

Participants aged ≥41 years had a lower likelihood than younger participants (18–20 years) to recognize ‘persistent pelvic pain’ (OR = 0.77, 95% CI: 0.61–0.97) (Table 4). On the other hand, women with high education were more likely than illiterate women to identify ‘persistent pelvic pain’. Additionally, women who were married, divorced, or widowed were more likely than single women to identify ‘unusual discomfort or pain during sex’. Participants who knew someone with cancer had a higher likelihood than participants who did not to recognize all warning signs with pain.

**Table 4:** Association between recognizing cervical cancer warning signs with pain and sociodemographic factors

Characteristic	Persistent pelvic pain			Unusual discomfort or pain during sex			Persistent lower back pain		
	n (%)	AOR (95% CI)^a^	***p***-value	n (%)	AOR (95% CI)^a^	***p***-value	n (%)	AOR (95% CI)^a^	***p***-value
**Age group**									
18 to 20	473 (11.3)	Ref	Ref	288 (8.7)	Ref	Ref	300 (10.2)	Ref	Ref
21 to 40	2627 (62.7)	0.85 (0.70–1.04)	0.12	2084 (63.0)	0.94 (0.77–1.15)	0.53	1818 (61.8)	1.01 (0.83–1.24)	0.91
41 or older	1088 (26.0)	0.77 (0.61–0.97)	0.025	936 (28.3)	0.95 (0.75–1.19)	0.64	823 (28.0)	1.09 (0.87–1.37)	0.47
**Educational level**									
Illiterate	56 (1.3)	Ref	Ref	58 (1.8)	Ref	Ref	55 (1.9)	Ref	Ref
Primary	200 (4.8)	1.14 (0.76–1.70)	0.54	168 (5.1)	0.81 (0.54–1.21)	0.30	165 (5.6)	0.92 (0.61–1.38)	0.68
Preparatory	593 (14.2)	1.44 (0.99–2.11)	0.06	482 (14.6)	0.96 (0.66–1.40)	0.84	398 (13.5)	0.80 (0.55–1.17)	0.25
Secondary	1343 (32.1)	1.62 (1.11–2.35)	0.011	1058 (32.0)	1.13 (0.78–1.64)	0.52	924 (31.4)	0.93 (0.64–1.36)	0.71
Diploma	452 (10.8)	1.80 (1.21–2.67)	0.004	337 (10.2)	1.12 (0.76–1.67)	0.57	308 (10.5)	0.98 (0.66–1.46)	0.92
Bachelor	1445 (34.5)	2.10 (1.43–3.08)	< 0.001	1133 (34.3)	1.54 (1.05–2.26)	0.027	1022 (34.8)	1.20 (0.81–1.76)	0.36
Postgraduate	99 (2.4)	3.06 (1.79–5.22)	< 0.001	72 (2.2)	1.64 (0.98–2.73)	0.06	69 (2.3)	1.44 (0.86–2.40)	0.16
**Occupation**									
Housewife	2702 (64.5)	Ref	Ref	2249 (68.0)	Ref	Ref	1932 (65.7)	Ref	Ref
Employed	920 (22.0)	1.04 (0.90–1.20)	0.61	709 (21.4)	0.99 (0.85–1.14)	0.86	635 (21.6)	0.93 (0.80–1.07)	0.32
Retired	24 (0.6)	0.40 (0.24–0.67)	0.001	28 (0.8)	0.87 (0.52–1.46)	0.59	20 (0.7)	0.58 (0.34–1.01)	0.053
Student	542 (12.9)	1.10 (0.88–1.36)	0.41	322 (9.7)	0.90 (0.73–1.12)	0.36	354 (12.0)	0.94 (0.76–1.17)	0.58
**Monthly income**									
< 1450 NIS	1394 (33.3)	Ref	Ref	1126 (34.0)	Ref	Ref	1007 (34.2)	Ref	Ref
≥ 1450 NIS	2794 (66.7)	1.04 (0.91–1.20)	0.57	2182 (66.0)	1.02 (0.89–1.17)	0.76	1934 (65.8)	1.00 (0.87–1.15)	0.98
**Residency**									
Gaza Strip	1592 (38.0)	Ref	Ref	1285 (38.8)	Ref	Ref	1144 (38.9)	Ref	Ref
WBJ	2596 (62.0)	0.95 (0.83–1.09)	0.45	2023 (61.2)	0.91 (0.80–1.04)	0.17	1797 (61.1)	0.91 (0.80–1.04)	0.18
**Having a chronic disease**									
No	3396 (81.1)	Ref	Ref	2657 (80.3)	Ref	Ref	2378 (80.9)	Ref	Ref
Yes	792 (18.9)	1.04 (0.91–1.19)	0.57	651 (19.7)	0.97 (0.85–1.12)	0.71	563 (19.1)	0.93 (0.81–1.07)	0.31
**Knowing someone with cancer**									
No	1642 (39.2)	Ref	Ref	1308 (39.5)	Ref	Ref	1163 (39.5)	Ref	Ref
Yes	2546 (60.8)	1.34 (1.21–1.48)	< 0.001	2000 (60.5)	1.22 (1.10–1.34)	< 0.001	1778 (60.5)	1.23 (1.11–1.35)	< 0.001
**Marital status**									
Single	983 (23.5)	Ref	Ref	581 (17.6)	Ref	Ref	662 (22.5)	Ref	Ref
Married	3009 (71.8)	1.24 (1.07–1.44)	0.005	2555 (77.2)	2.12 (1.82–2.46)	< 0.001	2122 (72.2)	1.15 (0.99–1.33)	0.07
Divorced	98 (2.3)	1.41 (0.99–2.01)	0.06	80 (2.4)	2.15 (1.53–3.04)	< 0.001	71 (2.4)	1.34 (0.95–1.89)	0.10
Widowed	98 (2.3)	1.18 (0.85–1.65)	0.33	92 (2.8)	2.23 (1.60–3.12)	< 0.001	86 (2.9)	1.39 (0.99–1.95)	0.053
**Site of data collection**									
Public spaces	1630 (38.9)	Ref	Ref	1240 (37.5)	Ref	Ref	1174 (39.9)	Ref	Ref
Hospitals	1086 (25.9)	0.99 (0.87–1.13)	0.89	915 (27.7)	1.04 (0.91–1.18)	0.56	713 (24.2)	0.82 (0.72–0.93)	0.002
Primary healthcare centers	1472 (35.1)	1.04 (0.92–1.17)	0.55	1153 (34.9)	0.92 (0.82–1.04)	0.20	1054 (35.8)	0.98 (0.87–1.10)	0.71

*AOR* adjusted odds ratio, *CI* confidence interval, *WBJ* West Bank and Jerusalem

^a^Adjusted for age-group, educational level, occupation, monthly income, marital status, residency, having a chronic disease, knowing someone with cancer, and site of data collection

### Recognizing CC warning signs of a non-specific nature

Women aged 21 to 40 years were less likely than younger women to recognize ‘persistent vaginal discharge that smells unpleasant’ (OR = 0.79, 95% CI: 0.64–0.96) (Table 5). On the contrary, women who had benefitted from higher education were more likely than illiterate women to recognize all warning signs of a nonspecific nature except ‘persistent diarrhea’ for which, no differences were found. Similarly, participants who knew someone with cancer had a higher likelihood than participants who did not get to know someone with cancer to identify all warning signs of a nonspecific nature except ‘persistent diarrhea’, where no differences were noticed. Married participants were more likely than single participants to identify ‘unexplained weight loss’ (OR = 1.37, 95% CI: 1.18–1.60) and ‘extreme generalized fatigue’ (OR = 1.41, 95% CI: 1.21–1.64).

**Table 5:** Association between recognizing cervical cancer warning signs of a non-specific nature and sociodemographic factors

Characteristic	Extreme generalized fatigue			Unexplained weight loss			Persistent vaginal discharge that smells un-pleasant			Persistent diarrhea		
	n (%)	AOR (95% CI)^a^	***p***-value	n (%)	AOR (95% CI)^a^	***p***-value	n (%)	AOR (95% CI)^a^	***p***-value	n (%)	AOR (95% CI)^a^	***p***-value
**Age group**												
18 to 20	488 (10.6)	Ref	Ref	499 (9.8)	Ref	Ref	346 (11.1)	Ref	Ref	139 (9.0)	Ref	Ref
21 to 40	2866 (62.3)	0.82 (0.67–1.01)	0.06	2815 (61.5)	0.99 (0.81–1.22)	0.94	1862 (59.6)	0.79 (0.64–0.96)	0.017	944 (60.9)	1.17 (0.91–1.49)	0.23
41 or older	1247 (27.1)	0.79 (0.62–0.99)	0.049	1314 (28.7)	1.03 (0.82–1.31)	0.79	915 (29.3)	0.94 (0.75–1.18)	0.62	468 (30.2)	1.17 (0.89–1.55)	0.27
**Educational level**												
Illiterate	62 (1.3)	Ref	Ref	67 (1.5)	Ref	Ref	45 (1.4)	Ref	Ref	37 (2.4)	Ref	Ref
Primary	245 (5.3)	1.39 (0.92–2.09)	0.11	276 (0.6)	1.69 (1.11–2.55)	0.014	181 (5.8)	1.48 (0.97–2.25)	0.07	113 (7.3)	1.10 (0.70–1.72)	0.69
Preparatory	683 (14.8)	1.64 (1.12–2.40)	0.011	726 (15.9)	1.76 (1.20–2.59)	0.004	446 (14.3)	1.39 (0.94–2.06)	0.10	257 (16.6)	0.91 (0.59–1.39)	0.66
Secondary	1509 (32.8)	1.93 (1.32–2.81)	0.001	1534 (33.5)	1.88 (1.29–2.74)	0.001	994 (31.8)	1.58 (1.07–2.32)	0.020	487 (31.4)	0.77 (0.50–1.17)	0.21
Diploma	483 (10.5)	2.10 (1.41–3.13)	< 0.001	461 (10.1)	1.58 (1.06–2.35)	0.025	312 (10.0)	1.50 (1.00–2.25)	0.052	161 (10.4)	0.74 (0.47–1.15)	0.18
Bachelor	1523 (33.1)	2.64 (1.79–3.88)	< 0.001	1429 (31.2)	1.87 (1.27–2.75)	0.002	1076 (34.5)	2.05 (1.38–3.05)	< 0.001	462 (29.8)	0.70 (0.45–1.07)	0.10
Postgraduate	96 (2.1)	3.30 (1.93–5.63)	< 0.001	85 (1.9)	1.78 (1.06–3.00)	0.031	69 (2.2)	2.25 (1.34–3.78)	0.002	34 (2.2)	0.84 (0.47–1.50)	0.56
**Occupation**												
Housewife	3131 (68.1)	Ref	Ref	3139 (68.6)	Ref	Ref	2049 (65.6)	Ref	Ref	1045 (67.4)	Ref	Ref
Employed	896 (19.5)	0.70 (0.60–0.81)	< 0.001	909 (19.9)	0.88 (0.76–1.02)	0.08	665 (21.3)	0.89 (0.77–1.03)	0.12	329 (21.2)	1.00 (0.84–1.19)	0.98
Retired	31 (0.7)	0.43 (0.26–0.71)	0.001	33 (0.7)	0.55 (0.33–0.92)	0.023	28 (0.9)	0.74 (0.45–1.24)	0.26	11 (0.7)	0.79 (0.40–1.56)	0.51
Student	543 (11.8)	0.87 (0.70–1.08)	0.21	497 (10.9)	0.87 (0.70–1.08)	0.20	381 (12.2)	0.88 (0.71–1.08)	0.23	166 (10.7)	0.90 (0.69–1.17)	0.43
**Monthly income**												
< 1450 NIS	1573 (34.2)	Ref	Ref	1579 (34.5)	Ref	Ref	1015 (32.5)	Ref	Ref	554 (35.7)	Ref	Ref
≥ 1450 NIS	3028 (65.8)	1.07 (0.93–1.24)	0.33	2999 (65.5)	1.03 (0.89–1.18)	0.74	2108 (67.5)	1.03 (0.90–1.18)	0.68	997 (64.3)	0.84 (0.71–0.99)	0.037
**Residency**												
Gaza Strip	1773 (38.5)	Ref	Ref	1759 (34.4)	Ref	Ref	1137 (36.4)	Ref	Ref	580 (37.4)	Ref	Ref
WBJ	2828 (61.5)	0.96 (0.84–1.11)	0.61	2819 (61.6)	0.94 (0.82–1.08)	0.42	1986 (63.6)	1.06 (0.93–1.21)	0.36	971 (62.6)	1.07 (0.91–1.25)	0.42
**Having a chronic disease**												
No	3692 (80.2)	Ref	Ref	3640 (79.5)	Ref	Ref	2484 (79.5)	Ref	Ref	1228 (79.2)	Ref	Ref
Yes	909 (19.8)	1.10 (0.96–1.27)	0.19	938 (20.5)	1.05 (0.91–1.21)	0.51	639 (20.5)	1.04 (0.91–1.19)	0.53	323 (20.8)	0.99 (0.84–1.16)	0.88
**Knowing someone with cancer**												
No	1830 (39.8)	Ref	Ref	1749 (38.2)	Ref	Ref	1228 (39.3)	Ref	Ref	682 (44.0)	Ref	Ref
Yes	2771 (60.2)	1.31 (1.18–1.45)	< 0.001	2829 (61.8)	1.51 (1.36–1.67)	< 0.001	1895 (60.7)	1.22 (1.11–1.35)	< 0.001	869 (56.0)	0.92 (0.82–1.04)	0.19
**Marital status**												
Single	981 (21.3)	Ref	Ref	940 (20.5)	Ref	Ref	709 (22.7)	Ref	Ref	323 (20.8)	Ref	Ref
Married	3399 (73.9)	1.41 (1.21–1.64)	< 0.001	3410 (74.5)	1.37 (1.18–1.60)	< 0.001	2247 (72.0)	1.14 (0.98–1.32)	0.09	1140 (73.5)	1.18 (0.98–1.41)	0.07
Divorced	109 (2.4)	1.91 (1.31–2.79)	0.001	101 (2.2)	1.41 (0.98–2.02)	0.06	81 (2.6)	1.59 (1.13–2.24)	0.008	38 (2.5)	1.19 (0.79–1.77)	0.41
Widowed	112 (2.4)	1.29 (0.92–1.82)	0.14	127 (2.8)	1.55 (1.08–2.21)	0.016	86 (2.8)	1.19 (0.86–1.67)	0.30	50 (3.2)	1.17 (0.79–1.72)	0.43
**Site of data collection**												
Public spaces	1665 (36.2)	Ref	Ref	1670 (36.5)	Ref	Ref	1219 (39.0)	Ref	Ref	700 (45.1)	Ref	Ref
Hospitals	1159 (25.2)	0.98 (0.86–1.11)	0.78	1237 (27.0)	1.05 (0.92–1.20)	0.47	809 (25.9)	0.95 (0.84–1.08)	0.41	369 (23.8)	0.62 (0.53–0.72)	< 0.001
Primary healthcare centers	1777 (38.6)	1.52 (1.33–1.72)	< 0.001	1671 (36.5)	1.14 (1.00–1.29)	0.045	1095 (35.1)	1.00 (0.89–1.13)	0.97	482 (31.1)	0.62 (0.53–0.71)	< 0.001

*AOR* adjusted odds ratio, *CI* confidence interval, *WBJ* West Bank and Jerusalem

^a^Adjusted for age-group, educational level, occupation, monthly income, marital status, residency, having a chronic disease, knowing someone with cancer, and site of data collection

### Good knowledge and its associated factors

Only 1934 participants (27.4%) had good knowledge of CC warning signs (Table 6). Participants from the Gaza Strip were slightly more likely than participants from the WBJ to have a good level of knowledge (29.7% vs 26.0%). The multivariable analysis identified factors associated with an increase in the odds of having good knowledge of CC warning signs, which were having a bachelor or postgraduate degree, being married, divorced, or widowed as well as knowing someone with cancer (Table 7). On the other hand, being employed or retired was associated with a decrease in the odds of having good knowledge.

**Table 6:** Knowledge level of cervical cancer warning signs

Level	Total n (%)	Gaza Strip n (%)	WBJ n (%)	***p***-value
**Poor**	1998 (28.3)	709 (26.7)	1289 (29.3)	0.002
**Fair**	3126 (44.3)	1158 (43.6)	1968 (44.7)	
**Good**	1934 (27.4)	788 (29.7)	1146 (26.0)	

*n* number of participants, *WBJ* West Bank and Jerusalem

**Table 7:** Association between having a good knowledge level of cervical cancer warning signs and sociodemographic factors

Characteristic	Good knowledge				
	n (%)	COR (95% CI)	***p***-value	AOR (95% CI)^a^	***p***-value
**Age group**					
18 to 20	161 (8.3)	Ref	Ref	Ref	Ref
21 to 40	1202 (62.2)	1.42 (1.18–1.71)	< 0.001	1.04 (0.82–1.31)	0.74
41 or older	571 (29.5)	1.51 (1.23–1.84)	< 0.001	1.19 (0.92–1.55)	0.19
**Educational level**					
Illiterate	30 (1.6)	Ref	Ref	Ref	Ref
Primary	115 (5.9)	1.26 (0.80–2.01)	0.32	1.18 (0.74–1.89)	0.49
Preparatory	280 (14.5)	1.15 (0.75–1.78)	0.51	1.08 (0.69–1.68)	0.74
Secondary	636 (32.9)	1.24 (0.82–1.89)	0.31	1.29 (0.84–1.99)	0.25
Diploma	187 (9.7)	1.04 (0.67–1.62)	0.85	1.22 (0.77–1.94)	0.39
Bachelor	642 (33.2)	1.28 (0.84–1.95)	0.25	1.60 (1.03–2.50)	0.038
Postgraduate	44 (2.3)	1.51 (0.88–2.61)	0.14	1.95 (1.09–3.46)	0.023
**Occupation**					
Housewife	1360 (70.3)	Ref	Ref	Ref	Ref
Employed	380 (19.6)	0.84 (0.73–0.96)	0.009	0.84 (0.72–0.99)	0.040
Retired	9 (0.5)	0.36 (0.18–0.73)	0.005	0.42 (0.20–0.88)	0.020
Student	185 (9.6)	0.66 (0.55–0.78)	< 0.001	0.91 (0.71–1.17)	0.47
**Monthly income**					
< 1450 NIS	691 (35.7)	Ref	Ref	Ref	Ref
≥ 1450 NIS	1243 (64.3)	0.89 (0.80–0.99)	0.045	1.01 (0.87–1.18)	0.87
**Marital status**					
Single	328 (17.0)	Ref	Ref	Ref	Ref
Married	1501 (77.6)	1.71 (1.49–1.96)	< 0.001	1.65 (1.38–1.97)	< 0.001
Divorced	49 (2.5)	1.89 (1.32–2.71)	0.001	1.95 (1.34–2.83)	0.001
Widowed	56 (2.9)	1.71 (1.22–2.38)	0.002	1.85 (1.27–2.68)	0.001
**Residency**					
Gaza Strip	788 (40.7)	Ref	Ref	Ref	Ref
WBJ	1146 (59.3)	0.83 (0.75–0.93)	0.001	0.88 (0.76–1.02)	0.09
**Having a chronic disease**					
No	1557 (80.5)	Ref	Ref	Ref	Ref
Yes	377 (19.5)	0.97 (0.85–1.11)	0.70	0.91 (0.78–1.05)	0.20
**Knowing someone with cancer**					
No	723 (37.4)	Ref	Ref	Ref	Ref
Yes	1211 (62.6)	1.29 (1.16–1.44)	< 0.001	1.29 (1.15–1.44)	< 0.001
**Site of data collection**					
Public Spaces	700 (36.2)	Ref	Ref	Ref	Ref
Hospitals	477 (24.7)	0.96 (0.84–1.10)	0.57	0.89 (0.77–1.03)	0.11
Primary healthcare centers	757 (39.1)	1.26 (1.11–1.42)	< 0.001	1.12 (0.99–1.28)	0.08

*COR* crude odds ratio, *AOR* adjusted odds ratio, *CI* confidence interval

^a^Adjusted for age-group, educational level, occupation, monthly income, marital status, residency, having a chronic disease, knowing someone with cancer, and site of data collection

*Note* The binary outcome of good knowledge was treated as a yes/no variable

## Discussion

The overall awareness of CC warning signs in this study was low. Participants from the Gaza Strip were slightly more likely than participants from the WBJ to have a good knowledge level. The factors associated with having good knowledge were having a bachelor or postgraduate degree, being married, divorced, or widowed as well as knowing someone with cancer. The most frequently identified warning sign was ‘vaginal bleeding after menopause’ followed by non-specific warning signs, namely ‘generalized fatigue’ and ‘unexplained weight loss’.

Awareness of CC warning signs is crucial for timely recognition and early seeking to medical advice in order to decrease CC-related mortality [5, 30, 31]. This study assessed the Palestinian women’s awareness level of CC warning signs to support the development of awareness-raising educational campaigns. This is especially essential in low-resource settings, where no prevention approaches and screening programs exist as in Palestine [13].

### Knowledge level of CC warning signs

Early CC detection, which is influenced by the level of awareness, remains one of the cornerstones of CC control strategies to improve survival rates in low- and middle-income countries [31–34]. In the absence of screening as well as HPV-vaccination programs, early detection and treatment of CC could be the most effective strategy to reduce resulting mortality and morbidity. Furthermore, multiple barriers to early presentation with cancer warning signs exist among Palestinian women, including financial restrictions, scarcity of female specialists, negative cancer beliefs, and paucity of treatment opportunities [12, 35–40]. Among these barriers, lack of knowledge and awareness is only one factor, but this one can be addressed by effective educational interventions [11, 41]. Low levels of knowledge of CC warning signs were also found by previous studies from the area of the Middle Eastern and North Africa, such as in Tunisia, Kuwait, Jordan, Qatar and Libya [27, 28, 42–44]. This may reflect poor health education about CC warning signs in Arab countries and underline the need for establishing continuous educational programs. A further contributing factor might be that the incidence of CC is relatively low in the Arab world, which leads to less experience and interest to learn more about this specific cancer [45–47].

In concordance with findings of this study, previous studies showed that women with higher levels of education were more likely to recognize CC warning signs [28, 29, 42, 43, 48–50]. This might be related to reading more about health-related topics and having a higher chance of working or meeting with people who similarly had good knowledge of health-related topics. Adlard and colleagues reported that knowing a family member or a friend who experienced cancer was associated with a higher awareness of cancer symptoms and warning signs [49]. This was consistent with the results of this study and with other studies that surveyed women in the United Kingdom [48, 49].

Married women in this study had a higher likelihood than single women to identify more CC warning signs, which was also noticed in previous studies conducted in the United Kingdom and China [48, 50]. Compared with single women, married women may be more concerned to read about topics related to their reproductive health and possibly have more opportunity to come across information when accessing maternity care or sexual and reproductive healthcare.

### Comparing knowledge between the Gaza strip and WBJ

Participants from the Gaza Strip were slightly more likely than participants from the WBJ to have a good level of knowledge and a higher likelihood to identify all warning signs with blood. The political situation in Palestine may play a role in this. In the WBJ, the fear of Israeli security forces’ harm and indignity at checkpoints may have created stress and avoidance of accessing healthcare services. This may also have limited women’s interaction with healthcare professionals and visitors to hospitals and clinics that can play a major role in shaping their knowledge level [51, 52].

The closures, barriers, and checkpoints can impact the daily life of Palestinians by adding hours of delay, unpredictability, and inability to seek medical advice and obtain health-related information and instructions. Women living in rural areas in the WBJ were reported to be the most challenged with these difficulties in accessing healthcare services, which negatively impacted their maternal health and chance to benefit from awareness initiatives [53, 54]. The interaction with social networks in the Palestinian community seems to have a key role in building good knowledge. This observation is based on the finding in this study that women who knew someone with cancer were more likely to have a good knowledge level.

Another contributing factor to the difference in knowledge between the Gaza Strip and the WBJ could be the proportion of women living in rural areas. There are more women living in rural areas in the WBJ, which may limit their access to internet and public libraries. This may have resulted in lower chances for the WBJ women to read more about health-related topics.

### Recognizing CC warning signs with blood vs other warning signs

In this study, warning signs with bleeding (including ‘irregular bleeding’, ‘unusual time’, and ‘unusual length or quantity’) were the most recognized warning signs of CC. This was also found in other studies conducted in Libya and the United Kingdom [28, 48]. However, ‘persistent vaginal discharge that smells unpleasant’ was less recognized than warning signs with bleeding or other non-specific warning signs. This differs from what was found among British women, where ‘persistent, abnormal or unusual vaginal discharge’ was more reported than ‘unexplained weight loss’ and ‘extreme generalized fatigue’ [48]. A possible reason for this could be that women’s thoughts of warning signs alarming them of the possibility of CC are influenced by the culture of the country where they were raised. In Palestine, it is common among women to believe that vaginal bleeding could be related more often to irregularities of the menstrual cycle. This may drive Palestinian women to read more about the possible causes of warning signs with bleeding; hence, they can have higher recognition of them as CC warning signs. On the other hand, women in high-income countries usually participate in educational health-activities related to sexually transmitted diseases especially during adolescence [55–57], which is not the case in Palestine. This could explain their ability to recognize unusual vaginal discharge more often than Palestinian women who did not acquire such knowledge because of the lack of similar educational programs.

Married women were more likely than single women to identify warning signs with blood and ‘unusual discomfort or pain during sex’. This was also observed in another study conducted in Qatar [43]. A possible explanation for this finding could be that single women in the Palestinian community are not usually sexually active and getting pregnant which may limit their interaction with obstetrics and gynecology clinics. In addition, single women possibly feel shy about reading or talking about CC warning signs they might experience. This is in comparison with married women who already had the opportunity to acquire knowledge from encountering similar problems overtime, hearing their friends’ or relatives’ stories, or through contact with healthcare professionals during their maternal visits.

### Future directions

The findings of this study underline the necessity to establish continuous educational programs that should focus on enriching Palestinian women’s knowledge of CC. Awareness campaigns are also needed and should be tailored to be appropriate for the specific cultural needs. Raising awareness of CC may make women feel more confident and encourage them to discuss their warning signs with healthcare professionals as soon as they recognize them. This will facilitate early detection and diagnosis of CC and improve patient prognosis.

### Strengths and limitations

The main strengths of this study include the use of a translated version of the validated tool (CeCAM) to assess women’s awareness of CC warning signs and the high response rate. In addition, the large sample size covering most geographical areas of Palestine and the stratified approach allowed direct measurement of knowledge about CC warning signs on different levels in the Palestinian community. This study also has some limitations. The use of stratified convenience sampling limits the generalizability of the findings. However, the large number of participants and the diversity of geographical areas covered in this study may mitigate this limitation. Another limitation could be that the study included participants who did not experience actual CC warning signs and looked at their perceived knowledge. Further research is needed to assess the awareness of women presented with CC warning signs and diagnosed with it afterwards.

## Conclusion

The overall knowledge of women included in this study was low with only 27.4% of women demonstrating a good level of knowledge of CC warning signs. Women residing in the Gaza Strip demonstrated a slightly better knowledge than women residing in the WBJ. The most frequently identified warning sign was ‘vaginal bleeding after menopause’ followed by ‘generalized fatigue’ and ‘unexplained weight loss’. The factors associated with having good knowledge of CC warning signs were having a bachelor or postgraduate degree, being married, divorced, or widowed as well as knowing someone with cancer. To increase women’s knowledge about CC warning signs, special health educational programs are needed.

## Supplementary Information

**Additional file 1.** Results of the bivariable analyses for the association between each category of CC warning signs and participant characteristics.

**Additional file 2.**

## Data Availability

The dataset used and analyzed during the current study is available from the corresponding author on reasonable request.

## Abbreviations

CC: Cervical cancer
HPV: Human papillomavirus
WBJ: West Bank and Jerusalem
PHCs: Primary healthcare centers
MoH: Ministry of health
CeCAM: Cervical cancer awareness measure
CI: Confidence interval
OR: Odds ratio
