# Supplementary material for: Knowledge of Palestinian women about cervical cancer warning signs: a national cross- sectional study
Source: BMC Public Health. 2021 Oct 2;21:1779. doi: 10.1186/s12889-021-11792-8 (PMC8487127; doi:10.1186/s12889-021-11792-8)
Supplement: Supplementary file 1 — Additional file 1. Results of the bivariable analyses for the association between each category of CC warning signs and participant characteristics. [file 12889_2021_11792_MOESM1_ESM.docx]

**Knowledge of Palestinian Women about Cervical Cancer Warning Signs: A National Cross- sectional Study**

Mohamedraed Elshami, MD, MMSc^1,2^*, Ibrahim Al-Slaibi, MD^3^*, Hanan Abukmail, MD^2,4^*, Mohammed Alser, MD^2^*, Afnan Radaydeh^5^, Alaa Alfuqaha^6^, Mariam Thalji^5^, Salma Khader^5^, Lana Khatib^7^, Noor Fannon, PharmB^8^, Bisan Ahmad^4^, Lina Kassab^2^, Hiba Khrishi^9^, Deniz Elhussaini^10^, Nour Abed^4^, Aya Nammari^5^, Tumodir Abdallah^5^, Zaina Alqudwa^10^, Shahd Idais^5^, Ghaid Tanbouz, DDS^9^, Ma'alem Hajajreh^11^, Hala Abu Selmiyh^4^, Zakia Abo-Hajouj^5^, Haya Hebi^5^, Manar Zamel^7^, Refqa Skaik^10^, Lama Hammoud^9^, Siba Rjoub^5^, Hadeel Ayesh^5^, Toqa Rjoub^5^, Rawan Zakout^4^, Amany Alser^12^, Nasser Abu-El-Noor, PhD^13#^, Bettina Bottcher, MD, PhD^4#^

*Contributed equally as a first co-author.

^#^Contributed equally as a senior co-author.

^1^Harvard Medical School, Boston, MA, USA.

^2^Ministry of Health, Gaza, Palestine.

^3^Almakassed Hospital, Jerusalem, Palestine.

^4^Faculty of Medicine, Islamic University of Gaza, Gaza, Palestine.

^5^Faculty of Medicine, Al-Quds University, Jerusalem, Palestine.

^6^Faculty of Graduate Studies, An-Najah National University, Nablus, Palestine.

^7^Faculty of Medicine, An-Najah National University, Nablus, Palestine.

^8^Faculty of Pharmacy, Alazhar University of Gaza, Gaza, Palestine.

^9^Faculty of Dentistry and Dental Surgery, Al-Quds University, Jerusalem, Palestine.
^10^Faculty of Medicine, Alazhar University of Gaza, Gaza, Palestine.

^11^Alia Hospital, Hebron, Palestine.

^12^Al-Shiffa hospital, Gaza, Palestine.

^13^Faculty of Nursing, Islamic University of Gaza, Gaza, Palestine.

**Corresponding author**

Mohamedraed Elshami, MD, MMSc

Harvard Medical School, 25 Shattuck Street, Boston, Massachusetts 02115

Phone: 832-245-6055

Email: mohamedraed.elshami@gmail.com

Supplement 1: Bivariable analysis to test the association between recognizing warning signs with blood and participant characteristics.

COR= crude odds ratio, CI= confidence interval, WBJ= West Bank and Jerusalem.

| **Characteristic** | **Vaginal bleeding after menopause** | | **Vaginal bleeding between periods** | | **Having menstrual periods that are heavier or longer than usual** | | **Vaginal bleeding during or after sex** | | **Blood in the stool or urine** | |
| --- | --- | --- | --- | --- | --- | --- | --- | --- | --- | --- |
|  | **COR (95% CI)** | **p-value** | **COR (95% CI)** | **p-value** | **COR (95% CI)** | **p-value** | **COR (95% CI)** | **p-value** | **COR (95% CI)** | **p-value** |
| **Age group**  18 to 20  21 to 40  41 or older | Ref  0.93 (0.79- 1.11)  0.87 (0.72- 1.04) | Ref  0.45  0.13 | Ref  0.89 (0.76- 1.04)  0.97 (0.82- 1.15) | Ref  0.16  0.75 | Ref  1.26 (1.07- 1.47)  1.46 (1.24- 1.73) | Ref  0.004  <0.001 | Ref  1.77 (1.51- 2.07)  1.72 (1.45- 2.04) | Ref  <0.001<0.001 | Ref  0.69 (0.59- 0.80)  0.60 (0.51- 0.72) | Ref  <0.001 <0.001 |
| **Educational level**  Illiterate  Primary  Preparatory  Secondary  Diploma  Bachelor  Postgraduate | Ref  1.28 (0.85- 1.91)  1.74 (1.20- 2.54)  1.93 (1.34- 2.77)  1.98 (1.35- 2.91)  2.12 (1.48- 3.05)  2.16 (1.29- 3.63) | Ref  0.23  0.004  <0.001  <0.001  <0.001  0.003 | Ref  1.26 (0.84- 1.87)  1.67 (1.15- 2.41)  1.70 (1.19- 2.44)  1.73 (1.19- 2.53)  1.97 (1.38- 2.83)  1.97 (1.20- 3.21) | Ref  0.26  0.007  0.004  0.004  <0.001  0.007 | Ref  1.30 (0.87- 1.94)  1.50 (1.04- 2.17)  1.39 (0.97- 1.98)  1.23 (0.84- 1.79)  1.32 (0.92- 1.89)  1.68 (1.03- 2.74) | Ref  0.20  0.030  0.07  0.29  0.13  0.039 | Ref  0.79 (0.53- 1.18)  1.15 (0.80- 1.66)  1.16 (0.81- 1.65)  1.03 (0.71- 1.50)  1.35 (0.94- 1.93)  2.09 (1.28- 3.44) | Ref  0.26  0.46  0.43  0.89  0.10  0.003 | Ref  0.93 (0.63- 1.39)  0.98 (0.68- 1.42)  1.16 (0.81- 1.66)  0.99 (0.68- 1.44)  1.28 (0.89- 1.83)  1.12 (0.69- 1.81) | Ref  0.74  0.93  0.41  0.96  0.18  0.65 |
| **Occupation**  Housewife  Employed  Retired  Student | Ref  0.82 (0.73- 0.94)  0.33 (0.21- 0.53)  1.00 (0.85- 1.18) | Ref  0.003  <0.001  0.96 | Ref  0.91 (0.81- 1.03)  0.52 (0.32- 0.83)  1.03 (0.88- 1.19) | Ref  0.14  0.007  0.74 | Ref  0.96 (0.86- 1.09)  0.38 (0.23- 0.62)  0.75 (0.65- 0.87) | Ref  0.54  <0.001  <0.001 | Ref  0.97 (0.86- 1.09)  0.58 (0.36- 0.94)  0.58 (0.50- 0.68) | Ref  0.62  0.027  <0.001 | Ref  1.12 (1.00- 1.26)  0.41 (0.24- 0.70)  1.50 (1.30- 1.74) | Ref  0.06  0.001  <0.001 |
| **Monthly income**  < 1450 NIS  ≥ 1450 NIS | Ref  0.82 (0.73- 0.92) | Ref  <0.001 | Ref  0.87 (0.78- 0.96) | Ref  0.005 | Ref  0.86 (0.79- 0.95) | Ref  0.003 | Ref  0.91 (0.83- 1.01) | Ref  0.07 | Ref  1.25 (1.13- 1.38) | Ref  <0.001 |
| **Residency**  Gaza Strip  WBJ | Ref  0.61 (0.55- 0.69) | Ref  <0.001 | Ref  0.68 (0.61- 0.75) | Ref  <0.001 | Ref  0.80 (0.72- 0.88) | Ref  <0.001 | Ref  0.80 (0.72- 0.88) | Ref  <0.001 | Ref  1.40 (1.27- 1.55) | Ref  <0.001 |
| **Having a chronic disease**  No  Yes | Ref  1.03 (0.90- 1.17) | Ref  0.70 | Ref  1.14 (1.01- 1.28) | Ref  0.035 | Ref  1.18 (1.05- 1.33) | Ref  0.007 | Ref  0.96 (0.85- 1.08) | Ref  0.50 | Ref  0.87 (0.78- 0.98) | Ref  0.023 |
| **Knowing someone with cancer**  No  Yes | Ref  1.35 (1.22- 1.51) | Ref  <0.001 | Ref  1.28 (1.16- 1.41) | Ref  <0.001 | Ref  1.19 (1.08- 1.31) | Ref  0.001 | Ref  1.20 (1.09- 1.32) | Ref  <0.001 | Ref  1.28 (1.17- 1.41) | Ref  <0.001 |
| **Marital status**  Single  Married  Divorced  Widowed | Ref  1.15 (1.02- 1.29)  1.15 (0.80- 1.66)  0.87 (0.63- 1.19) | Ref  0.026  0.46  0.39 | Ref  1.16 (1.03- 1.30)  0.93 (0.67- 1.30)  0.93 (0.68- 1.25) | Ref  0.011  0.68  0.62 | Ref  1.44 (1.29- 1.61)  1.55 (1.10- 2.17)  1.55 (1.14- 2.11) | Ref  <0.001  0.012  0.005 | Ref  2.16 (1.93- 2.43)  2.32 (1.66- 3.25)  1.86 (1.37- 2.51) | Ref  <0.001  <0.001  <0.001 | Ref  0.74 (0.66- 0.83)  1.25 (0.89- 1.75)  0.71 (0.53- 0.96) | Ref  <0.0010.20  0.028 |
| **Site of data collection**  Public spaces  Hospitals  Primary healthcare centers | Ref  0.85 (0.75- 0.97)  1.02 (0.91- 1.16) | Ref  0.013  0.70 | Ref  0.83 (0.73- 0.93)  1.10 (0.98- 1.23) | Ref  0.002  0.11 | Ref  0.94 (0.83- 1.06)  1.14 (1.02- 1.27) | Ref  0.29  0.025 | Ref  1.08 (0.96- 1.21)  1.19 (1.07- 1.33) | Ref  0.22  0.002 | Ref  0.70 (0.62- 0.78)  0.87 (0.78- 0.98) | Ref  <0.001  0.016 |

Supplement 2: Bivariable analysis to test the association between recognizing warning signs with pain and participant characteristics.

| **Characteristic** | **Persistent pelvic pain** | | **Unusual discomfort or pain during sex** | | **Persistent lower back pain** | |
| --- | --- | --- | --- | --- | --- | --- |
|  | **COR (95% CI)** | **p-value** | **COR (95% CI)** | **p-value** | **COR (95% CI)** | **p-value** |
| **Age group**  18 to 20  21 to 40  41 or older | Ref  0.92 (0.79- 1.08)  0.74 (0.62- 0.88) | Ref  0.32  0.001 | Ref  1.51 (1.29- 1.77)  1.47 (1.24- 1.74) | Ref  <0.001  <0.001 | Ref  1.10 (0.94- 1.29)  1.09 (0.92- 1.29) | Ref  0.24  0.33 |
| **Educational level**  Illiterate  Primary  Preparatory  Secondary  Diploma  Bachelor  Postgraduate | Ref  1.21 (0.81- 1.81)  1.60 (1.10- 2.31)  1.79 (1.25- 2.57)  1.83 (1.25- 2.67)  2.25 (1.57- 3.22)  3.22 (1.93- 5.36) | Ref  0.34  0.013  0.001  0.002  <0.001  <0.001 | Ref  0.83 (0.56- 1.24)  0.99 (0.68- 1.43)  1.02 (0.71- 1.46)  0.93 (0.64- 1.36)  1.19 (0.83- 1.71)  1.30 (0.80- 2.10) | Ref  0.36  0.94  0.92  0.73  0.33  0.29 | Ref  0.89 (0.59- 1.32)  0.78 (0.54- 1.14)  0.88 (0.62- 1.27)  0.88 (0.60- 1.29  1.08 (0.75- 1.55)  1.31 (0.81- 2.12) | Ref  0.55  0.20  0.50  0.51  0.68  0.28 |
| **Occupation**  Housewife  Employed  Retired  Student | Ref  1.19 (1.06- 1.34)  0.38 (0.23- 0.63)  1.20 (1.04- 1.40) | Ref  0.004  <0.001  0.015 | Ref  0.99 (0.88- 1.11)  0.73 (0.45- 1.18)  0.63 (0.54- 0.73) | Ref  0.81  0.20  <0.001 | Ref  1.06 (0.94- 1.19)  0.57 (0.34- 0.97)  0.97 (0.84- 1.13) | Ref  0.33  0.037  0.70 |
| **Monthly income**  < 1450 NIS  ≥ 1450 NIS | Ref  1.07 (0.97- 1.18) | Ref  0.20 | Ref  0.99 (0.89- 1.09) | Ref  0.81 | Ref  0.97 (0.88- 1.08) | Ref  0.60 |
| **Residency**  Gaza Strip  WBJ | Ref  0.96 (0.87- 1.06) | Ref  0.41 | Ref  0.91 (0.82- 0.99) | Ref  0.045 | Ref  0.91 (0.83- 1.00) | Ref  0.06 |
| **Having a chronic disease**  No  Yes | Ref  0.87 (0.78- 0.98) | Ref  0.025 | Ref  0.99 (0.88- 1.11) | Ref  0.82 | Ref  0.93 (0.83- 1.05 | Ref  0.25 |
| **Knowing someone with cancer**  No  Yes | Ref  1.34 (1.21- 1.48) | Ref  <0.001 | Ref  1.24 (1.12- 1.36) | Ref  <0.001 | Ref  1.22 (1.11- 1.35) | Ref  <0.001 |
| **Marital status**  Single  Married  Divorced  Widowed | Ref  1.01 (0.90- 1.13)  1.20 (0.85- 1.69)  0.74 (0.55- 0.99) | Ref  0.91  0.30  0.049 | Ref  1.89 (1.69- 2.12)  2.00 (1.44- 2.79)  1.76 (1.30- 2.38) | Ref  <0.001  <0.001  <0.001 | Ref  1.09 (0.97- 1.22)  1.29 (0.92- 1.79)  1.25 (0.93- 1.70) | Ref  0.15  0.14  0.14 |
| **Site of data collection**  Public spaces  Hospitals  Primary healthcare centers | Ref  0.88 (0.78- 0.99)  0.96 (0.86- 1.07) | Ref  0.040  0.48 | Ref  1.10 (0.98- 1.24)  1.02 (0.92- 1.14) | Ref  0.11  0.66 | Ref  0.78 (0.70- 0.89)  0.96 (0.86- 1.07) | Ref  <0.001  0.50 |

COR= crude odds ratio, CI= confidence interval, WBJ= West Bank and Jerusalem.

COR= crude odds ratio, CI= confidence interval, WBJ= West Bank and Jerusalem.

| **Characteristic**  Supplement 3: Bivariable analysis to test the association between recognizing warning signs of a nonspecific nature and participant characteristics. | **Extreme generalized fatigue** | | **Unexplained weight loss** | | **Persistent vaginal discharge that smells un-pleasant** | | **Persistent diarrhea** | |
| --- | --- | --- | --- | --- | --- | --- | --- | --- |
|  | **COR (95% CI)** | **p-value** | **COR (95% CI)** | **p-value** | **COR (95% CI)** | **p-value** | **COR (95% CI)** | **p-value** |
| **Age group**  18 to 20  21 to 40  41 or older | Ref  1.07 (0.91- 1.26)  0.95 (0.79- 1.13) | Ref  0.39  0.53 | Ref  1.27 (1.08- 1.49)  1.37 (1.15- 1.63) | Ref  0.003  <0.001 | Ref  0.89 (0.77- 1.04)  1.03 (0.87- 1.21) | Ref  0.16  0.76 | Ref  1.24 (1.02- 1.51)  1.38 (1.12- 1.71) | Ref  0.035  0.003 |
| **Educational level**  Illiterate  Primary  Preparatory  Secondary  Diploma  Bachelor  Postgraduate | Ref  1.57 (1.05- 2.34)  1.88 (1.30- 2.72)  2.02 (1.41- 2.89)  1.79 (1.23- 2.61)  2.16 (1.51- 3.10)  2.40 (1.45- 3.96) | Ref  0.028  0.001  <0.001  0.003  <0.001  0.001 | Ref  1.86 (1.24- 2.79)  1.92 (1.33- 2.79)  1.81 (1.26- 2.59)  1.35 (0.93- 1.97)  1.54 (1.07- 2.20)  1.44 (0.88- 2.34) | Ref  0.003  0.001  0.001  0.12  0.019  0.15 | Ref  1.45 (0.96- 2.19)  1.32 (0.90- 1.93)  1.39 (0.96- 2.02)  1.25 (0.85- 1.85)  1.65 (1.14- 2.40)  1.82 (1.11- 2.98) | Ref  0.08  0.16  0.08  0.26  0.008  0.017 | Ref  0.93 (0.60- 1.44)  0.77 (0.52- 1.16)  0.66 (0.44- 0.97)  0.65 (0.43- 0.99)  0.62 (0.42- 0.93)  0.80 (0.46- 1.37) | Ref  0.74  0.22  0.037  0.043  0.020  0.41 |
| **Occupation**  Housewife  Employed  Retired  Student | Ref  0.75 (0.66- 0.84)  0.39 (0.25- 0.64)  0.81 (0.70- 0.95) | Ref  <0.001  <0.001  0.007 | Ref  0.77 (0.68- 0.87)  0.44 (0.27- 0.71)  0.65 (0.56- 0.75) | Ref  <0.001  0.001  <0.001 | Ref  1.04 (0.92- 1.17)  0.87 (0.53- 1.40)  1.00 (0.86- 1.15) | Ref  0.52  0.56  0.96 | Ref  0.99 (0.86- 1.14)  0.65 (0.34- 1.25)  0.82 (0.68- 0.98) | Ref  0.87  0.20  0.031 |
| **Monthly income**  < 1450 NIS  ≥ 1450 NIS | Ref  0.96 (0.87- 1.07) | Ref  0.47 | Ref  0.93 (0.84- 1.03) | Ref  0.15 | Ref  1.12 (1.01- 1.23) | Ref  0.028 | Ref  0.90 (0.80- 1.01) | Ref  0.09 |
| **Residency**  Gaza Strip  WBJ | Ref  0.89 (0.81- 0.99) | Ref  0.029 | Ref  0.91 (0.82- 1.00) | Ref  0.06 | Ref  1.10 (0.99- 1.21) | Ref  0.06 | Ref  1.01 (0.90- 1.14) | Ref  0.84 |
| **Having a chronic disease**  No  Yes | Ref  0.99 (0.88- 1.12) | Ref  0.92 | Ref  1.13 (1.00- 1.28) | Ref  0.046 | Ref  1.08 (0.96- 1.21) | Ref  0.21 | Ref  1.09 (0.94- 1.25) | Ref  0.25 |
| **Knowing someone with cancer**  No  Yes | Ref  1.29 (1.17- 1.43) | Ref  <0.001 | Ref  1.53 (1.38- 1.69) | Ref  <0.001 | Ref  1.25 (1.14- 1.38) | Ref  <0.001 | Ref  0.96 (0.85- 1.08) | Ref  0.47 |
| **Marital status**  Single  Married  Divorced  Widowed | Ref  1.41 (1.26- 1.58)  1.67 (1.16- 2.39)  1.00 (0.74- 1.36) | Ref  <0.001  0.005  0.99 | Ref  1.58 (1.41- 1.77)  1.45 (1.03- 2.06)  1.56 (1.14- 2.15) | Ref  <0.001  0.034  0.006 | Ref  1.07 (0.96- 1.20)  1.48 (1.07- 2.07)  1.12 (0.83- 1.51) | Ref  0.24  0.019  0.48 | Ref  1.20 (1.05- 1.38)  1.35 (0.92- 1.99)  1.49 (1.05- 2.10) | Ref  0.009  0.13  0.025 |
| **Site of data collection**  Public spaces  Hospitals  Primary healthcare centers | Ref  0.98 (0.87- 1.11)  1.58 (1.40- 1.78) | Ref  0.75  <0.001 | Ref  1.16 (1.03- 1.31)  1.28 (1.14- 1.43) | Ref  0.016  <0.001 | Ref  0.91 (0.80- 1.02)  0.96 (0.86- 1.07) | Ref  0.10  0.49 | Ref  0.69 (0.60- 0.80)  0.69 (0.60- 0.79) | Ref  <0.001  <0.001 |
