# Supplementary material for: Knowledge of Palestinian women about cervical cancer warning signs: a national cross- sectional study
Source: BMC Public Health. 2021 Oct 2;21:1779. doi: 10.1186/s12889-021-11792-8 (PMC8487127; doi:10.1186/s12889-021-11792-8)
Supplement: Supplementary file 2 — Additional file 2. [file 12889_2021_11792_MOESM2_ESM.docx]

**Knowledge of Cervical Cancer Warning Signs Questionnaire**

Serial number: ………. Location: …………. Governorate: ……….

1. **Sociodemographic Data**

- Age: …….. years
- Marital status: 🞏 Single 🞏 Married 🞏 Divorced 🞏 Widowed
- Highest level of education: 🞏 Illiterate 🞏 Primary 🞏 Prep 🞏 Secondary

🞏 Diploma 🞏 Bachelor degree 🞏 Postgraduate

- Occupation: 🞏 Housewife 🞏 Employed 🞏 Retired 🞏 Still studying
- Monthly income (NIS): ………………
- Do you have any chronic disease? 🞏 No 🞏 Yes
- Have you, your family or close friends had cancer? 🞏 No 🞏 Yes

1. **Confidence and Recognition of Cervical Cancer Warning Signs**

- How confident are you that you would notice a cervical cancer warning sign?

🞏 Not at all confident 🞏 Not very confident 🞏 Fairly confident 🞏 Very confident

| Table (3): The following may or may not be warning signs for cervical cancer. We are interested in your opinion: | | | | | |
| --- | --- | --- | --- | --- | --- |
| Warning Sign | **1= Strongly disagree** | **2= Disagree** | **3= Not sure** | **4= Agree** | **5= Strongly agree** |
| 1. Do you think vaginal bleeding between periods could be a sign of cervical cancer? |  |  |  |  |  |
| 1. Do you think persistent lower back pain could be a sign of cervical cancer? |  |  |  |  |  |
| 1. Do you think a persistent vaginal discharge that smells unpleasant could be a sign of cervical cancer? |  |  |  |  |  |
| 1. Do you think discomfort or pain during sex could be a sign of cervical cancer? |  |  |  |  |  |
| 1. Do you think menstrual periods that are heavier or longer than usual could be a sign of cervical cancer? |  |  |  |  |  |
| 1. Do you think persistent diarrhea could be a sign of cervical cancer? |  |  |  |  |  |
| 1. Do you think vaginal bleeding after the menopause could be a sign of cervical cancer? |  |  |  |  |  |
| 1. Do you think persistent pelvic pain could be a sign of cervical cancer? |  |  |  |  |  |
| 1. Do you think vaginal bleeding during or after sex could be a sign of cervical cancer? |  |  |  |  |  |
| 1. Do you think blood in the stool or urine could be a sign of cervical cancer? |  |  |  |  |  |
| 1. Do you think unexplained weight loss could be a sign of cervical cancer? |  |  |  |  |  |
| 1. Do you think that extreme fatigue could be a sign of cervical cancer? |  |  |  |  |  |
